# Supplementary material for: Tai Chi increases functional connectivity and decreases chronic fatigue syndrome: A pilot intervention study with machine learning and fMRI analysis
Source: PLoS One. 2022 Dec 1;17(12):e0278415. doi: 10.1371/journal.pone.0278415 (PMC9714925; doi:10.1371/journal.pone.0278415)
Supplement: S1 Table — (PDF) [file pone.0278415.s001.pdf]

S1 Table. The 60 Features after Features Selected by Linear SVC Model.

Number	ROI ID	ROI to ROI		Number	ROI ID	ROI to ROI	
1	V5838	Vis14_L	SomMot8_R	31	V84524	Vis11_R	Limbic-TempPole6_L
2	V8367	Vis20_L	Default-Temp1_R	32	V95102	SomMot7_R	SalVentAttn-FrOperIns1_R
3	V10939	Vis27_L	Cont-PFCl5_L	33	V95164	SomMot7_R	Default-Par3_R
4	V28566	DorsAttn-Post3_L	Default-PFC1_L	34	V102598	SomMot26_R	Default-pCunPCC9_L
5	V32978	DorsAttn-Post14_L	Default-PFC13_L	35	V106296	SomMot35_R	SalVentAttn-TempOccPar3_R
6	V34100	DorsAttn-Post17_L	SalVentAttn-FrOperIns4_L	36	V106297	SomMot35_R	SalVentAttn-TempOccPar4_R
7	V35111	DorsAttn-FEF2_L	SalVentAttn-Med1_R	37	V107496	SomMot38_R	SalVentAttn-TempOccPar3_R
8	V35112	DorsAttn-FEF2_L	SalVentAttn-Med2_R	38	V111125	DorsAttn-Post7_R	Limbic-TempPole1_R
9	V35969	DorsAttn-FEF4_L	Default-Temp3_R	39	V118266	SalVentAttn-TempOccPar2_R	SomMot36_R
10	V36591	DorsAttn-PrCv2_L	Default-pCunPCC2_L	40	V118269	SalVentAttn-TempOccPar2_R	SomMot39_R
11	V37498	SalVentAttn-ParOper2_L	SalVentAttn-TempOccPar5_R	41	V118302	SalVentAttn-TempOccPar2_R	SalVentAttn-FrOperIns1_R
12	V39323	SalVentAttn-FrOperIns2_L	Limbic-TempPole5_L	42	V118666	SalVentAttn-TempOccPar3_R	SomMot36_R

13	V39686	SalVentAttn-FrOperIns3_L	DorsAttn-FEF1_L	43	V118894	SalVentAttn-TempOccPar4_R	SalVentAttn-ParOper3_L
14	V39747	SalVentAttn-FrOperIns3_L	Cont-Cing2_L	44	V120638	SalVentAttn-PrC1_R	SomMot8_R
15	V39958	SalVentAttn-FrOperIns3_L	Cont-Cing1_R	45	V124088	SalVentAttn-PFCl1_R	DorsAttn-FEF3_L
16	V45759	Limbic-OFC1_L	Default-Par1_L	46	V124488	SalVentAttn-Med1_R	DorsAttn-FEF3_L
17	V48582	Limbic-TempPole3_L	Default-PFC17_L	47	V129878	Limbic-OFC6_R	DorsAttn-Post8_R
18	V48899	Limbic-TempPole4_L	SalVentAttn-FrOperIns3_L	48	V129936	Limbic-OFC6_R	Cont-Par5_R
19	V55228	Cont-PFCl4_L	Vis28_L	49	V129937	Limbic-OFC6_R	Cont-Par6_R
20	V55754	Cont-PFCl5_L	Default-Temp6_L	50	V134325	Cont-Par4_R	Limbic-TempPole1_R
21	V56379	Cont-PFCl6_L	Default-PFCdPFCm1_R	51	V134725	Cont-Par5_R	Limbic-TempPole1_R
22	V58500	Cont-Cing1_L	SalVentAttn-FrOperIns4_L	52	V141788	Cont-PFCl14_R	Default-PFC23_L
23	V61340	Default-Temp5_L	Cont-PFCl6_L	53	V142900	Cont-pCun2_R	SalVentAttn-FrOperIns4_L
24	V62358	Default-Temp7_L	Cont-Cing1_R	54	V142956	Cont-pCun2_R	Default-Temp8_L
25	V63315	Default-Temp10_L	Limbic-OFC2_L	55	V145438	Default-Par2_R	SomMot8_R
26	V66072	Default-Par7_L	DorsAttn-Post4_L	56	V146421	Default-Par5_R	Vis21_L

27	V69585	Default-PFC8_L	Default-PFCdPFCm7_R	57	V147290	Default-Temp2_R	DorsAttn-PrCv1_L
28	V70883	Default-PFC12_L	DorsAttn-Post15_L	58	V151341	Default-PFCv4_R	Cont-PFCl7_L
29	V76092	Default-pCunPCC1_L	SalVentAttn-ParOper1_L	59	V153774	Default-PFCdPFCm6_R	Default-PFC9_L
30	V79057	Default-pCunPCC8_L	SomMot27_R	60	V159164	Default-pCunPCC6_R	Default-Par3_R

Note: Because the Schaefer template has 400 ROIs, so it has 160000 functional connections (V1-V160000). The ROI ID represents the rank number of the functional connection, and the ROI to ROI represents each ROI name of the functional connection in the Schaefer template. However, it should be mentioned that this ROI to ROI haven't direction.
