# Supplementary material for: Tai Chi increases functional connectivity and decreases chronic fatigue syndrome: A pilot intervention study with machine learning and fMRI analysis
Source: PLoS One. 2022 Dec 1;17(12):e0278415. doi: 10.1371/journal.pone.0278415 (PMC9714925; doi:10.1371/journal.pone.0278415)
Supplement: S2 Table — (PDF) [file pone.0278415.s002.pdf]

S2 Table. Features Importance Permutation Test Result.

Time	ROI ID	ROI Name	Average score	Standard Deviation
1	V32978	ROI082-178	0.05976	0.06094869
1	V37498	ROI093-298	0.05014	0.04933539
1	V45759	ROI114-159	-0.00247	0.01083509
1	V48582	ROI121-182	-0.02555	0.05246616
1	V95164	ROI237-364	-0.02732	0.03862147
1	V102598	ROI256-198	-0.02472	0.05718323
1	V142900	ROI357-100	0.00465	0.02135129
1	V142956	ROI357-156	0.066	0.06533758
1	V146421	ROI366-021	-0.02721	0.05686929
2	V8367	ROI020-367	0.08325	0.07884122
2	V28566	ROI071-166	0.04961	0.07044393
2	V35969	ROI089-369	0.05056	0.03359593
2	V48899	ROI122-099	0.01001	0.0200075
2	V145438	ROI363-238	0.00215	0.03353919
3	V39323	ROI098-123	-0.0104	0.0255703
3	V45759	ROI114-159	-0.08526	0.07720578
3	V55754	ROI139-154	-0.0208	0.03494796
3	V70883	ROI177-083	0.00036	0.06120352

3	V124488	ROI311-088	-0.0701	0.06626455
4	V39323	ROI098-123	0.05035	0.05839844
4	V45759	ROI114-159	0.05169	0.07645027
4	V63315	ROI158-115	0.06897	0.08779886
4	V142956	ROI357-156	0.03098	0.04302603
4	V153774	ROI384-174	0.00751	0.03170331
4	V159164	ROI397-364	0.00281	0.02930194
5	V35111	ROI087-311	-0.0256	0.06206964
5	V35969	ROI089-369	0.02534	0.07873299
5	V63315	ROI158-115	0.0506	0.07788864
5	V76092	ROI190-092	-0.04971	0.05940047
5	V95164	ROI237-364	0.01251	0.04888763
6	V39686	ROI099-086	0.01025	0.02579608
6	V63315	ROI158-115	0.13336	0.08103771
6	V76092	ROI190-092	-0.0249	0.04431693
6	V111125	ROI277-325	-0.05007	0.03353349
6	V118266	ROI295-266	-0.03538	0.02274325
6	V145438	ROI363-238	0.20028	0.0877378
7	V63315	ROI158-115	0.02887	0.06500018
7	V70883	ROI177-083	0.09073	0.07347494

7	V134725	ROI336-325	0.07447	0.05545917
7	V142956	ROI357-156	0.01965	0.04344396
8	V10939	ROI027-139	-0.04542	0.02613472
8	V32978	ROI082-178	-0.03825	0.07042327
8	V70883	ROI177-083	-0.00572	0.07870376
8	V151341	ROI378-141	-0.04013	0.01990184
9	V8367	ROI020-367	0.07998	0.05705436
9	V39323	ROI098-123	0.05962	0.06249364
9	V55228	ROI138-028	0.04245	0.04795308
9	V66072	ROI165-072	-0.01385	0.07197692
9	V159164	ROI397-364	-0.07324	0.07045497
10	V28566	ROI071-166	0.00514	0.0341406
10	V84524	ROI211-124	0.04395	0.06941828
10	V141788	ROI354-188	0.09579	0.07106881
10	V147290	ROI368-090	0.12008	0.07616294

Note: Because the Schaefer template has 400 ROIs, so it has 160000 functional connections (V1-V160000). The ROI ID represents the rank number of the functional connection, and the ROI name represents the ROI number in the Schaefer template.
