# Supplementary material for: Tai Chi increases functional connectivity and decreases chronic fatigue syndrome: A pilot intervention study with machine learning and fMRI analysis
Source: PLoS One. 2022 Dec 1;17(12):e0278415. doi: 10.1371/journal.pone.0278415 (PMC9714925; doi:10.1371/journal.pone.0278415)
Supplement: S3 Table — (PDF) [file pone.0278415.s003.pdf]

S3 Table. Changes on features after the intervention.

Features	ROI ID	CFS		HC	
		t value	P value	t value	P value
1	V5838	-0.004	0.997	-0.776	0.447
2	V8367	1.450	0.163	-0.925	0.367
3	V10939	1.405	0.176	0.105	0.917
4	V28566	0.890	0.384	-1.821	0.084
5	V32978	2.809	0.011*	-2.238	0.037*
6	V34100	-0.430	0.672	1.275	0.218
7	V35111	-0.997	0.331	0.883	0.388
8	V35112	-0.594	0.560	0.107	0.916
9	V35969	2.504	0.022*	-2.449	0.024*
10	V36591	3.387	0.003*	-0.509	0.616
11	V37498	-1.460	0.161	-0.117	0.908
12	V39323	-2.655	0.016*	-0.983	0.338
13	V39686	-0.430	0.672	1.275	0.218
14	V39747	1.160	0.260	-1.492	0.152
15	V39958	0.690	0.499	-1.467	0.159
16	V45759	-1.245	0.228	1.646	0.116
17	V48582	-1.452	0.163	1.199	0.245

18	V48899	-2.655	0.016*	-0.983	0.338
19	V55228	1.405	0.176	0.105	0.917
20	V55754	2.238	0.037*	-0.982	0.338
21	V56379	0.369	0.716	-0.916	0.371
22	V58500	1.160	0.260	-1.492	0.152
23	V61340	2.238	0.037*	-0.982	0.338
24	V62358	-2.774	0.012*	1.228	0.234
25	V63315	-1.245	0.228	1.646	0.116
26	V66072	0.890	0.384	-1.821	0.084
27	V69585	-2.713	0.014*	-0.855	0.403
28	V70883	2.809	0.011*	-2.238	0.037*
29	V76092	3.387	0.003*	-0.509	0.616
30	V79057	2.193	0.041*	-1.033	0.314
31	V84524	1.751	0.096	-1.151	0.264
32	V95102	-1.590	0.128	0.480	0.637
33	V95164	2.634	0.016*	-0.279	0.783
34	V102598	2.193	0.041*	-1.033	0.314
35	V106296	-0.399	0.694	-0.661	0.516
36	V106297	-2.059	0.053	-0.518	0.610
37	V107496	-1.473	0.157	0.105	0.917

38	V111125	1.618	0.122	-1.120	0.277
39	V118266	-0.399	0.694	-0.661	0.516
40	V118269	-1.473	0.157	0.105	0.917
41	V118302	-2.334	0.031*	0.276	0.786
42	V118666	-2.059	0.053	-0.518	0.610
43	V118894	-1.460	0.161	-0.117	0.908
44	V120638	-1.590	0.128	0.480	0.637
45	V124088	-0.997	0.331	0.883	0.388
46	V124488	-0.594	0.560	0.107	0.916
47	V129878	1.618	0.122	-1.120	0.277
48	V129936	1.072	0.297	-1.498	0.151
49	V129937	0.991	0.334	-0.418	0.681
50	V134325	1.072	0.297	-1.498	0.151
51	V134725	0.991	0.334	-0.418	0.681
52	V141788	1.920	0.070	-1.326	0.201
53	V142900	0.690	0.499	-1.467	0.159
54	V142956	-2.774	0.012*	1.228	0.234
55	V145438	2.634	0.016*	-0.279	0.783
56	V146421	1.450	0.163	-0.925	0.367
57	V147290	2.504	0.022*	-2.449	0.024*

58	V151341	0.369	0.716	-0.916	0.371
59	V153774	-2.713	0.014∗	-0.855	0.403
60	V159164	-2.415	0.026∗	0.711	0.486

Note: Because the Schaefer template has 400 ROIs, so it has 160000 functional connections (V1-V160000). The ROI ID represents the rank number of the functional connection. The ∗ represents $P < 0.05$. Thus, CFS has 20 features with ∗, while HC has 4 features with ∗.
