# Supplementary material for: Tai Chi increases functional connectivity and decreases chronic fatigue syndrome: A pilot intervention study with machine learning and fMRI analysis
Source: PLoS One. 2022 Dec 1;17(12):e0278415. doi: 10.1371/journal.pone.0278415 (PMC9714925; doi:10.1371/journal.pone.0278415)
Supplement: S4 Table — (PDF) [file pone.0278415.s004.pdf]

S4 Table The score of the prediction accuracy in each repeat time.

**Repeat Time**	**Prediction Accuracy**
1	0.85
2	0.70
3	0.85
4	0.90
5	0.70
6	0.95
7	0.70
8	0.85
9	0.80
10	0.75

Note: The prediction accuracy represents that using the random forest model to predict in test data.
