# Supplementary figures and images for: Tai Chi increases functional connectivity and decreases chronic fatigue syndrome: A pilot intervention study with machine learning and fMRI analysis
Source: PLoS One. 2022 Dec 1;17(12):e0278415. doi: 10.1371/journal.pone.0278415 (PMC9714925; doi:10.1371/journal.pone.0278415)

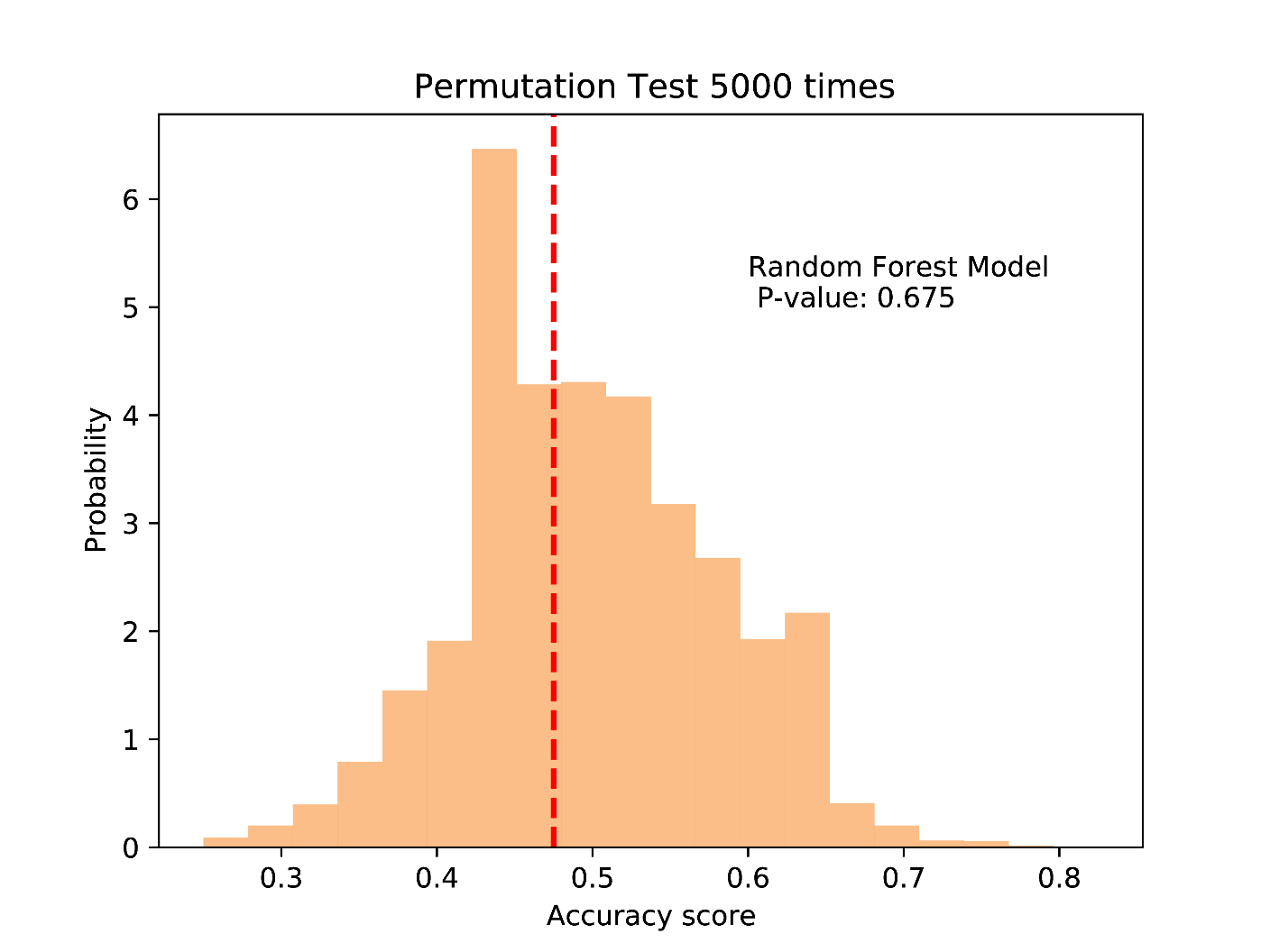

Supplement: S1 Fig — (TIF) [file pone.0278415.s005.tif]
