# Supplementary material for: Tai Chi increases functional connectivity and decreases chronic fatigue syndrome: A pilot intervention study with machine learning and fMRI analysis
Source: PLoS One. 2022 Dec 1;17(12):e0278415. doi: 10.1371/journal.pone.0278415 (PMC9714925; doi:10.1371/journal.pone.0278415)
Supplement: S1 File — (PDF) [file pone.0278415.s009.pdf]

Supplementary Analysis

We performed the statistical analysis based on Linear Mixed Effects Model (LMEM) for supplementary description to detect the interaction effects. These consisted of three parts, the analysis parameters, the LMEM results of DMN network and the LMEM results of left FPN.

LMEM Analysis Parameters

Repeated covariance type was compound symmetry. Covariates included gender, age, head motion and BMI. Fixed factors included group (CFS group and healthy group), time (pre-intervention and post-intervention) and the interaction of group and time. Random effect was the intercept of each subject. Estimation method was maximum likelihood. The multiple comparisons method was Bonferroni.

The LMEM results of the DMN

The null presumption was that the group and time and their interaction effect were unconnected with the functional connections changes of the DMN within 60 features found by machine learning.

Results showed the AIC of this model was 269.550. The type III tests of fixed effects showed the group was significant (F = 18.795, P = 0.000), the time was insignificant (F = 0.027, P = 0.870), and the interaction of group and time was significant (F = 10.887, P = 0.002). The pairwise comparisons of simple effect analysis showed that, when the time was controlled, the patient group versus the healthy group was significant (mean difference = -2.048, P = 0.000, 95% CI = [-2.808, -1.289]) in the pre-intervention, and was insignificant (mean difference = -0.059, P = 0.878, 95% CI = [-0.818, 0.701]) in the post-intervention. When the group was controlled, the pre-intervention versus the post-intervention was significant (mean difference = -0.945, P = 0.028, 95% CI = [-1.785, -0.105]) in the CFS group,

and was also significant (mean difference = 1.045, $P = 0.016$, 95% CI = [0.205, 1.885]) in the healthy group.

The LMEM results of the left FPN

The null presumption was that the group and time and their interaction effect were unconnected with the functional connections changes of the left FPN within 60 features found by machine learning.

Results showed the AIC of this model was 213.349. The type Ⅲ tests of fixed effects showed the group was significant ($F = 5.349$, $P = 0.026$), the time was insignificant ($F = 0.407$, $P = 0.527$), and the interaction of group and time was significant ($F = 10.131$, $P = 0.003$). The pairwise comparisons of simple effect analysis showed that, when the time was controlled, the patient group versus the healthy group was significant (mean difference = -1.026, $P = 0.001$, 95% CI = [-1.595, -0.458]) in the pre-intervention, and was insignificant (mean difference = -0.109, $P = 0.704$, 95% CI = [-0.677, 0.460]) in the post-intervention. When the group was controlled, the pre-intervention versus the post-intervention was significant (mean difference = -0.551, $P = 0.010$, 95% CI = [-0.963, -0.139]) in the CFS group, and was insignificant (mean difference = 0.367, $P = 0.079$, 95% CI = [-0.045, 0.779]) in the healthy group.

In summary, under the pre-assumption of the DMN / left FPN network, both of them displayed a significance in the interaction effect of the group and time. Further, after multiple comparisons correction, CFS group and healthy group both showed a significance in the simple effect analysis between interventions on the DMN network ($P_{CFS} = 0.028$ and $P_{healthy} = 0.016$). However, CFS group showed a significance in the simple effect analysis between interventions on the left FPN network, while healthy group hadn't ($P_{CFS} = 0.010$ and $P_{healthy} = 0.079$). But when considered that the 60 features

included seven networks, all of their *P* value were insignificant (single network: $\alpha = 0.05 / 7 = 0.0071$; bilateral network: $\alpha = 0.05 / 14 = 0.0036$).
