# Supplementary material for: Tai Chi increases functional connectivity and decreases chronic fatigue syndrome: A pilot intervention study with machine learning and fMRI analysis
Source: PLoS One. 2022 Dec 1;17(12):e0278415. doi: 10.1371/journal.pone.0278415 (PMC9714925; doi:10.1371/journal.pone.0278415)
Supplement: Attachment — Submitted filename: Response to Reviewers.docx [file pone.0278415.s010.docx]

**Cover Letter**

Dear Editor,

We appreciate you and all the reviewers for your precious time in reviewing our paper and providing valuable comments. It was your valuable and insightful comments that led to possible improvements in the current version. The authors have carefully considered the comments and tried our best to address every one of them, and a final revised version was both submitted. We hope the manuscript after careful revisions and specialized polishing meets your high standards. The authors welcome further constructive comments if any.

Besides, please update our financial disclosure with ‘The funders had no role in study design, data collection and analysis, decision to publish, or preparation of the manuscript’. We find that we misunderstood about financial disclosure, sorry for that.

We have revised the manuscript format to meet the PLOS ONE’s requirements, please check.

Sincerely,

Yours

Kang Wu

Dongzhimen Hospital, Beijing University of Chinese Medicine

Beijing, China

**Response to Review 1**

**Comment 1:** The statistical analysis is somewhat simplistic, but serves the purpose. An alternative, potentially more coherent analysis would have been to use a linear mixed effects model framework to assess the interaction of treatment and time, and to evaluate the various contrasts of interest (potentially with more power).

**Response:** Thanks for your kind reminders. We used the linear mixed effects model analysis, and its results can be found in the supplementary file (S1 File in Supporting Information).

As you expected, the interaction effect of the treatment and time was significant (*P* = 0.002). However, on the pairwise comparisons of simple effect analysis, the FC changes between interventions in CFS group (*P* = 0.028) and healthy group (*P* = 0.016) were significant in the single large-scale network (α = 0.05) level, but not the whole network (α = 0.05/7 = 0.007) level. Thus, the linear mixed effects model analysis was not selected.

To avoid the random errors in the individual level, **we performed the covariance analysis on inter-group comparison, taking gender, age, BMI and head motion as the confound matrix, instead of the independent student t test in before**. As were showed in Table 2 and Table 3, the results of covariance analysis were very similar to before. [Line 272, Line 280, Pg 12-13].

**Comment 2:** Can the authors remark on whether the functional connections (FCs) that the authors found can be defined in a new subject? Or, are these only able to be retrospectively defined? How would they be measured in a new subject?

**Response:** Thanks for your kind reminders. The FCs can be found in every subject beyond our trial, as long as the researcher performs the same processes with us.

To repeat our outcome, three parts should be kept. Part 1 is to use fmriprep software. Part 2 is to select Schaefer template to extract the time series. Part 3 is to find the time series of the 60 regions in the Schaefer template.

All the information about these 60 regions could be found in Supporting Information (S1 Table) and our github repository, including the label, name, MNI space coordination of each region. Also, the Schaefer template and the predictive model were uploaded together.

Our github repository address https://github.com/Clancy-wu/TaiChi-CFS-2022.

**Comment 3:** With regard to the limitations of the study, given the amount of data collected, is it possible that even an equally-sized set of completely random data would yield a similar number of FCs? This is an issue in large-scale genomics studies.

**Response:** Thanks for your kind reminders. As you said, the large-scale network analysis is to find result within a large number of data, but we do not think this is a trick in mathematic.

First, using FCs to make prediction has been reported in before. About 12 years ago, the author Dosenbach accomplished 91% accuracy to make prediction via functional connections between regions of brain networks, and published article in the top journal SCIENCE (Dosenbach, 2010, *Science*, doi:10.1126/science.1194144). This means the process ‘predict via FCs with brain network’ is a trustworthy method.

Second, many robust steps were performed to make results reliable in our trial. In pre-process, we used a high-repeatability software ‘fmriprep’ to ensure that the time series we got is reliable. In statistical analysis, we used the strict multiple-correction method Bonferroni to ensure the result reliable. A random data cannot pass the multiple correction in such a strict level (α = 0.05/7 = 0.007 and α = 0.05/14 = 0.0036).

Third, our results fitted previous literature. Our outcomes focus on two networks, the DMN and left FPN. As we discussed in article, the DMN network was the most reported network in CFS patients in previous (three studies reported among seven studies in CFS area). And the FPN, especially the left, was found to work in physical exercise (including Tai Chi practice) via a meta-analysis (Yu Qian, *Brain structure & function*, 2021). Therefore, our result is an extension of the previous finding, not an irrelevantly random innovation with other studies.

To sum up, we think our finds within the amount of data in the article is much meaningful, and these cannot be replaced by the equally-sized random data.

**Comment 4:** Line 53: This statement about the relationship of Tai Chi to the broader area of Qigong is not necessarily universally held. It may be better to say that in some lineages of Tai Chi and with some teachers, there can be substantial overlap with practices found in Qigong. I note that in many cases, taijiquan is taught with a completely different emphasis and with completely different defining characteristics, especially if the goal is martial usage of jin and qi.

**Response:** Thanks for your kind reminders. We changed our description and re-wrote this paragraph [Line 52-64, Pg 3].

**Comment 5:** Line 60: Again, it is quite debatable whether Tai Chi is a representative Qigong. This also ignores the issue of whether the state defined simplified 24-style is an adequate representation of taijiquan in general. And, obviously, the teacher has the primary effect in terms of whether a student can actually learn the skills required for qigong or taijiquan.

**Response:** Thanks for your kind reminders. We changed our description and re-wrote this paragraph [Line 52-64, Pg 3].

**Comment 6:** Line 77: Change to "could be a mechanism".

**Response:** Thanks for your kind reminders. We realized that we are not rigorous enough in word, your carefulness helps us a lot. We revised this paragraph.

**Comment 7:** Line 85: This absolute statement about "traditional statistical [sic] methods" is quite overblown. In fact, many statistical techniques make relatively few assumptions or relatively mild assumptions. It can just as easily be said that most machine learning techniques suffer from a complete lack of theoretical underpinning, interpretability, or generalizability.

**Response:** Thanks for your kind reminders. Sorry for that we exaggerated the criticism for traditional statistical method to present the machine learning. We changed our description [Line 95-97, Pg 5].

**Comment 8:** Line 98: What would be the mechanism of action for improving the special functional connectivity?

**Response:** Thanks for your kind reminders. Sorry for that our wrong expression misled you. We revised our description [Line 107-108, Pg 5]:

**Comment 9:** Line 130: The phrase "was unclear" implies partial knowledge even though full knowledge was expected. Do the authors mean "blinded" here?

**Response:** Thanks for your kind reminders. Sorry for that our wrong expression misled you. We used ‘blinded’ to replace ‘unclear’ [Line 142, Pg 7].

**Comment 10:** Line 204: Why was the max-min normalization performed? It seems unnecessary given the statistical methods used.

**Response:** Thanks for your kind reminders. The SF-36 questionnaire contains eight parts — Physical Functioning, Role-Physical, Bodily Pain, General Health, Vitality, Social Functioning, Role-Emotional, Mental Health — and each part calculates differently with others.

For instance, the score of Physical Functioning equals (real score -10) / 20 * 100; the score of Role-Physical equals (real score - 4) / 4 * 100, etc.

The ‘max-min normalization’ represents the calculation of the score of each part via their own formula. The ‘average score ’ represents summing up scores of eight parts and then dividing eight (Table 1) [Line 242, Pg 11].

Therefore, we could use one value to show the average health statement of each subject, and to analyze the improvement of Tai Chi for the health statement (displayed in Table 1) via covariance analysis.

**Comment 11:** Line 313: "[M]achine learning is better at prediction than statistical inference" is likely a truism because machine learning pays nearly no attention to the issues of statistical inference.

**Response:** Thanks for your kind reminders. Sorry for our poor expression. We revised this sentence ‘machine learning only plays role in prediction rather than statistical inference’ [Line 383-384, Pg 18].

**Comment 12:** Line 347: Does the phrase "at the bottom of my life" correctly interpret the author's intent?

**Response:** Thanks for your kind reminders. Sorry for that my poor expression worried you. I revised this sentence ‘that they gave me much care in my trough time’ [Line 419, Pg 20]. Thanks a lot.

**Comment 13:** Figure 2: Change "Liner" to "Linear". Also, clarify that feature construction was performed only on training data.

**Response:** Thanks for your kind reminders. We revised Fig 2.

**Response to Review 2**

**Comment 1:** Line 102: There is no power analysis, to define sample size. I would like to see a power analysis to be sure whether the sample size is enough or not for the fMRI analysis. Why do you choose 20 patients and 20 healthy control?

**Response:** Thanks for your kind reminders. Speaking honestly, we haven’t provided power analysis, because our sample size was defined by the median of previous literature. Despite the small sample size, we want to make explanations for our trial in two parts, why we choose 20:20 samples and whether this sample size could be enough to show our result.

Question 1: Why we choose 20:20 samples?

(1) In fMRI area, the cost of fMRI data acquisition for one subject is much expensive than other clinical trials. Shortly ago, Scott Marek published an article in *Nature*, saying that the sample with a good replication rate in fMRI analysis requires over thousands (Marek, S., *Nature*, 2022), which caused quite a bit of controversy. Of course, the larger the sample size, the more convincing the results are. However, to accomplish such a great sample size is very difficult.

In year 2017, Russell A. Poldrack obtained 548 studies in fMRI area at year 2011-2015, found that the median group size was 19 subjects in single group (Poldrack, R. A., *Nature reviews. Neuroscience*, 2017). In year 2020, Russell A Poldrack again pointed out, result from literature review showed that more than half of the samples comprised fewer than 50 people, in the machine learning & fMRI area (Poldrack, R. A., *JAMA psychiatry*, 2020). From those we can see, our trial samples size 20:20 was at the median level compared with the previous studies.

(2) Patients with CFS disease are hard to recruit. In the area of CFS and machine learning as well as fMRI, there were three previous studies (Provenzano et al., 2020; Sevel et al., 2018; Provenzano et al., 2020), two enrolled 38 CFS patients (both belongs to the author Provenzano), and one enrolled 18 CFS patients (author Sevel). Compared with them, our study enrolled 20 CFS patients, ranks the second. This also means that our trial was at the median level.

Taking those two parts into consideration, we defined the sample size of the trial into 20:20, and we think, under the median level sample size, our trial result could discover some things reliable, like the previous literature.

Question 2: Whether this sample size could be enough to show our result?

Under this median level sample size, we made efforts to increase the reliable and repeatability of our trial result in two parts.

(1) We are a longitudinal trial in CFS & machine learning & fMRI area.

Scott Marek — the author we mentioned in Question 1 — highly recommends the intervention trial design with fMRI, and importantly, he doesn’t deny the value of the longitudinal design in the small-sample neuroimaging. He said in his Nature article “Within-person designs (for example, **longitudinal**) … or both (for example, **interventions**) frequently have increased measurement reliability and effect sizes … Thus, small-sample neuroimaging will always be critical for studying the human brain”. In fact, the previous three studies in CFS & machine learning & fMRI area (Provenzano et al., 2020; Sevel et al., 2018; Provenzano et al., 2020) were all were cross-sectional designs. And our trial could be the first longitudinal design in this study area. Thus, compared to them, whether in the size of dataset or the difficulty to accomplish the trial, our experiment is more valuable, as well as the result more meaningful.

(2) We performed a series of robust technology to ensure the repeatability of the results.

We applied the unified fmriprep workflow to process the dataset, the five-fold cross-validation to evaluate model performance, the strict multiple-correction Bonferroni method, and 10 repetitions to compute each result. All of these increased the repeatability of our trial as much as possible.

In summary, under the median level sample size and the robust calculation processes, we believe our results of the trial are reliable and worthy to be published.

**Comment 2:** Line 138: MRI recording parameters have been given really weak. The structural (T1w) and functional recording parameters should be more clear and more details in terms of repeatability.

**Response:** Thanks for your kind reminders. We filled up the details of MRI recording parameters [Line 152-158, Pg 7]:

‘the resting-state echo-planar imaging sequence acquisition (time of repetition = 2,000 ms, time of echo = 30 ms, flip angle = 90°, phase encoding direction = A >> P, coverage = whole brain including cerebellum, field of view = 240 mm × 240 mm, matrix = 64 × 64, slice thickness = 3.5 mm, volumes = 240), the three-dimensional structure imaging adopting T1W1 sequence (time of repetition = 1900 ms, time of echo = 2.53 ms, coverage = whole brain including cerebellum, field of view = 250 mm × 250 mm, matrix = 256 × 256, slice thickness = 1.0 mm, volumes = 176)’.

**Comment 3:** ICA-AROMA tool is only capable of denoise movement artifacts. But the other artifacts such as respiratory, cardiac, or MRI device-based noise remain intact in resting-state data. Did the authors remove artifacts also manually? If they did not it should be written as a limitation.

**Response:** Thanks for your kind reminders. We add this into our limitation [Line 402-403, Page 19]:

‘Forth, although the denoise movement artifacts were removed, the other artifacts such as respiratory, cardiac and MRI device-based noise might remain influences to our data analysis.’

**Comment 4:** Authors use linear SVC for feature ranking, and then the most informative features are used to train a Random Forest classifier. The authors must justify why they did not perform classification with SVC. Besides, the feature ranking by SVC is based on having maximum accuracy by the SVC classifier. Can we guarantee that the same features will give the maximum classification result by the Random Forest? Since they already started with SVC we would like to see the classification results with SVC.

**Response:** Thanks for your kind reminders. We used linear SVC for features selection (decreased 160004 features into 60 features), and Random Forest classifier for prediction and features importance ranking (figure out which feature is important under 60 features). The workflow can be found in Fig 2.

Since our design is a longitudinal trial, not a cross-section, we would pay more attention to find that which brain areas work to the intervention, rather than to seek for highest accuracy. Generally speaking, the more features remained, the higher accuracy model would get, especially in cross-section trial. On the contrary, the target of our trial is to reduce the features number while maintaining a good accuracy as much as possible. This is the reason why we should perform ‘feature selection’ process.

There are many methods for feature selection. In our trial, we chose a method named ‘Select From Model’ (referred to scikit-learn software document), the Model can apply tree-based model or linear model. Here, we used linear SVC model for feature selection.

However, when the model is used for feature selection, it cannot be applied for classification in the meantime. The causing reason is that if the feature number is greatly reduced, the accuracy of the model will definitely decrease. For instance, in our trial, on the 160004 features, the linear SVC model we trained reached near 90% accuracy, but on the 60 features, it sharply reduced to half, because the model has been suited to the structure of 160004 features.

Consequently, we used two model, the linear SVC is used for features selection (in train data) and the random forest is used for classification (in train data and test data). The linear SVC tell us which features related to the intervention and the random forest verified prediction ability of these features.

We choose to use linear SVC because this model often has a good performance in machine learning with wide application. We choose to use random forest because this model always has a good performance in neuro-image area.

**Comment 5:** It would be better if we can see a more detailed comparative analysis of feature ranking and classification processes. For example (Line 224), they selected 60 of more than 160000 features. How the threshold value of 60 was determined? It would be great if we can see a plot of a number of most informative features vs obtained classification accuracy.

**Response:** Thanks for your kind reminders. We added the default threshold of features selection [Line 196, Pg 9], ‘…threshold = 1e-5…’.

From the document of scikit-learn software description, the default threshold of ‘Select From Model’ is 1e-5. We controlled feature selection via adjusting L1 value, thus forgot to explain the default threshold. Now we added.

As was said in Comment 4, the process of feature selection (a number of most informative feature) cannot apply the same model with the process of prediction (obtained classification accuracy). Feature selection (‘Select From Model’ method) is not a continues process that you can observe the feature number reduction step by step. Once we defined the L1 value, several features will be left, the L1 value size is irrelevant with the number of features left. We only can control L1 value from small to big, but the number of features will up and down without regular. Thus, the X-axis is hard to plot.

The obtained classification accuracy is generated by random forest model. When we got the selected features from the linear SVC (the most informative features), its initial performance was not good under the default random forest model, 50% - 60% actually. Then, we should tune the hyper-parameters of the random forest to increase accuracy. This largely depended on the researcher experience to know whether it really reach the best accuracy. You may cannot promise the best accuracy on each time of the most informative features you get.

Therefore, the plot is hard to make. The result we found in our trial was tried for thousands calculation. As we mentioned in the limitation, we may not have found all the important features, we just found a good unit of features and they predicted greater than before.

**Comment 6:** Line 186: Since the dataset is small, the cross-validation is wise to apply, but can we guarantee that 10 repeated runs would be theoretically enough to see the actual performance? A mathematical justification of this value “10” would be required. A plot of average accuracy vs. the number of repetitions would be helpful as well.

**Response:** Thanks for your kind reminders. We used five-fold cross-validation and repeated five-fold cross-validation on 10 times. In our trial, we performed five-fold cross-validation on training data for 10 repeats, we predicted on test data for 10 repeats, and applied permutation test on features ranking for 10 repeats. Actually, number 10 is not a value verified by mathematic, it is just generated by the conventional thinking. But for more credibility, we used permutation test to support the prediction accuracy score. We believe the score of permutation test and score of prediction on 10 repeats can complement each other to illustrate the performance of the random forest model, rather than relying on a certain score.

If only one score should be chosen, the score of permutation test will be more reliable.

We added the result of the prediction accuracy per time on S4 Table, and ROC score of the random forest model on S4 Fig.

**Response to Review 3**

**Comment 1:** The main hypotheses of this paper are very general. The introduction cites some studies about the effects of Tai Chi in general, but the authors just say that machine learning will find some “special pattern” of FC differences in CFS, and that the regions/networks/connections showing differences at baseline will show some kind of non-specific changes after the training. While the authors discuss some of the particular changes in the context of the literature in their Discussion, the Introduction would be improved by at least including some more specific background information about which functional networks may be affected in CFS, and why Tai Chi may change those networks.

**Response:** Thanks for your kind reminders. We added more details in the introduction and revised the paragraph [Line 81-89, Pg 4].

**Comment 2:** In the “Clinical design and evaluation” section, the authors state that a clinical evaluator was “unclear about the grouping of subjects in the study.” Does this mean they were blinded? Or something else? If there was no waitlist or other control group and everyone received the same Tai Chi intervention, what were the evaluators unclear about?

**Response:** Thanks for your kind reminders. We revised this sentence [Line 141-144, Pg 7]:

‘All subjects underwent clinical evaluation and resting-state fMRI scanning from the scale evaluator, who was blinded about the grouping of subjects in the study. The decision that the subject who entered the CFS group was made by clinician through consultation’.

In our trial, the clinical doctor decided the group of each subject, and another person (a postgraduate in our team) was responsible for scale evaluation. The evaluator may guess the subject group, but he would not know the answer. So, we think the evaluator could be said ‘blinded’.

**Comment 3:** The authors state that “subjects were required to practice Tai Chi for 30 minutes per day by themselves at home.” Do the authors have data to report about adherence to this requirement (i.e., how much home practice was completed by each participant)? It then says “the Tai Chi teaching and family Tai Chi exercise of each subject were recorded, including live recording…” Does this mean that each home practice session was recorded in some way as well, or just the group or instructor-led sessions? Please clarify.

**Response:** Thanks for your kind reminders. We revised this sentence [Line 137-140, Page 6]:

‘During our trial, for each subject, the Tai Chi teaching was recorded by live recording, and the family Tai Chi exercise was recorded through video feedback and telephone follow-up, in order to supervise the quality of the practice. In the end, all subjects had completed the required exercise time’

**Comment 4:** The data acquisition parameters require more details and clarification. No anatomical scan is mentioned, only stating the “parameters of this machine scan.” The parameters reported appear to be for a functional EPI sequence, but this is not specified. Also, while the time of repetition is stated as 2mm, the total number of volumes (and the total duration of the scan) is not reported.

**Response:** Thanks for your kind reminders. We filled up the details of MRI recording parameters [Line 152-158, Pg 7]:

‘The fMRI data were acquired by a Siemens 3-T MRI scanner (Germany), and the parameters of this machine scan were as follows: the resting-state echo-planar imaging sequence acquisition (time of repetition = 2,000 ms, time of echo = 30 ms, flip angle = 90°, phase encoding direction = A >> P, coverage = whole brain including cerebellum, field of view = 240 mm × 240 mm, matrix = 64 × 64, slice thickness = 3.5 mm, volumes = 240), the three-dimensional structure imaging adopting T1W1 sequence (time of repetition = 1900 ms, time of echo = 2.53 ms, coverage = whole brain including cerebellum, field of view = 250 mm × 250 mm, matrix = 256 × 256, slice thickness = 1.0 mm, volumes = 176)’.

**Comment 5:** The data preprocessing section states that smoothing was done at 2mm FWHM. This is much lower than us conventionally used. Typical guidelines suggest to use a FWHM equivalent to 2 or 3 times the size of the voxels. With a voxel size of 3.75x3.75x5mm as stated in the data acquisition section, we would expect a spatial smoothing kernel of at least 7-8mm FWHM. This step can have a significant impact on functional connectivity findings. Can the authors explain this unconventional preprocessing choice?

**Response:** Thanks for your kind reminders. We feel much sorry for this description mistake. The actual steps are that, voxel size normalized to 2 mm, and the FWHM for volume is [6 6 6]. This sentence has been revised [Line 167-168, Line 171, Pg 8]:

‘… voxel size normalizing to 2 mm… smoothing with Gaussian kernel of full width at half maximum of 6 mm’.

The 2 mm volume size and [6 6 6] of FWHM are the default setting in DPABISurf software and are recommended by Professor Chaogan Yan, who is the author of DPABISurf software.

**Comment 6:** In the Discussion, the words “decrease” and “lower” are used interchangeably, leading to some confusion as to whether it is referring to the assessment of baseline differences between CFS and healthy volunteers or to the changes resulting from the intervention. For instance, the sentence “Our trial found a similar decrease in the DMN” is confusing. The “trial” should refer to the Tai Chi intervention, but earlier the authors state that increased DMN FC was found following the intervention. So when discussing these results, the authors should clarify the distinction between the baseline differences and the changes observed following the trial/intervention.

**Response:** Thanks for your kind reminders. We adjusted our word description, using ‘lower’ on baseline and ‘decrease’ on the effect of the intervention.

**Comment 7:** The discussion could be improved with more interpretation of the results related to the DMN (i.e. the post-intervention increase in DMN connectivity). The authors currently say that DMN is important and “plays a central role in healthy people and patients with various diseases”, but do not elaborate much. They mention a previous hypothesis of DMN underconnectivity in CFS patients, which suggests that DMN underconnectivity is associated with greater energy needs and reduced energy available to the individual. They say that Tai Chi could “improve the abnormal pattern of the DMN,” but they do not elaborate on this point. This seems like potentially the most important part of the discussion, so it would be good to see this result discussed more. They go on to highlight that increased inter-network DMN connectivity was found after the intervention, and state that this is “interesting”. This could be improved by a couple sentences/references explaining why this might be interesting and worthy of further investigation. The discussion of FPN-DMN connectivity that follows is great!

**Response:** Thanks for your kind reminders. We added more details about DMN and FPN-DMN in the Discussion [Line 337-353, Pg 16-17].

**Comment 8:** The axis labels for Figure 6A and 6B say “differences” but this could be clarified by stating whether this is post-pre or pre-post. What are the units of these differences?

**Response:** Thanks for your kind reminders. We revised figure 6 and added a striking label ‘Post- minus Pre-’. Besides, we revised the caption of figure 6 [Line 306-307, Page 15]:

However, the unit cannot be clarified, because they haven’t been defined any unit in the previous literature, especially in the value of the functional connection.

Thanks very much for your attention and time. Looking forward to hearing from you.

Sincerely,

Yours

Kang Wu

Dongzhimen Hospital, Beijing University of Chinese Medicine

Beijing, China
