# Supplementary material for: Tai Chi increases functional connectivity and decreases chronic fatigue syndrome: A pilot intervention study with machine learning and fMRI analysis
Source: PLoS One. 2022 Dec 1;17(12):e0278415. doi: 10.1371/journal.pone.0278415 (PMC9714925; doi:10.1371/journal.pone.0278415)
Supplement: Attachment — Submitted filename: Response to Reviewers.docx [file pone.0278415.s011.docx]

**Cover Letter**

Dear Editor,

We appreciate you and all the reviewers for your precious time in reviewing our paper and providing valuable comments. It was your valuable and insightful comments that led to possible improvements in the current version. The authors have carefully considered the comments and tried our best to address every one of them, and a final revised version was both submitted. The authors welcome further constructive comments if any.

Besides, please update our financial disclosure with ‘The funders had no role in study design, data collection and analysis, decision to publish, or preparation of the manuscript’.

Sincerely,

Yours

Kang Wu

Dongzhimen Hospital, Beijing University of Chinese Medicine

Beijing, China

**Response to Review 3**

**Comment 1**: The authors have added a paragraph to help set up some of their hypotheses about how Tai Chi is expected to change functional connectivity patterns in CFS patients. However, I do still have some concerns about the relevance and clarity of this paragraph.

Even if few intervention studies have been done on CFS with neural outcomes, are there any studies showing what the baseline neural correlates of CFS are? What are the mechanisms by which Tai Chi would act on these neural signatures of CFS?

The following is not a complete sentence: “While others studies indicated that Tai Chi could improve sleep disorders through increasing functional connections of the sub-regions of DMN, including the medial prefrontal cortex and the medial temporal lobe [38,39].”

**Response:** Thanks for your kind reminders.

The neural baselines of CFS were descripted before the paragraph you mentioned, including follows **[Paragraph 3, Line 73-77]:**

(a) “Charles [30] detected the decreased intrinsic connectivity within the left frontoparietal network (LFPN) and the decreased extrinsic connectivity involving the sensory motor network (SMN) and the salience network (SN) on CFS patients, both of which were related to the level of their fatigue symptom”, the “decreased intrinsic connectivity” and “decreased extrinsic connectivity” indicated the neural baselines of CFS.

(b) “Besides, the default mode network (DMN), which is focused on recently, showed connection disruptions in CFS [29,31] and other fatigue related disease [32]”, the “connection disruptions” indicated the neural baseline of CFS.

The effects of Tai Chi on brain networks and brain areas could be found as follows **[Line 83-88]:**

“A study illustrated the effect of Tai Chi in regulating the rest-state functional connections of the DMN and LFPN to enhance cognitive function on healthy people [37]” and “others studies indicated that Tai Chi could improve sleep disorders through increasing functional connections of the sub-regions of DMN, including the medial prefrontal cortex and the medial temporal lobe [38,39]”, the “DMN” and “LFPN” are the brain networks that Tai Chi acts with, and the “prefrontal cortex” and “medial temporal lobe” are the specific brain areas that Tai Chi works with.

Combining the above, we now add a sentence to clarify our purpose “It seemed that functions of Tai Chi and disfunctions of CFS were overlapped partly in brain networks, for instance, the DMN and LFPN [Line 87-88]”.

In this article, we hypothesis that the reorganization of brain network’s functional connectivity maybe the mechanism of Tai Chi treating CFS. Thus, which brain networks were participated in this reorganization is the study target of our research majorly. However, the mechanisms of why Tai Chi could change these brain networks and how Tai Chi made such a changed pattern were beyond this article.

Besides, thanks for your carefulness. We apologize for this sentence mistake. The sentence was revised as follows:

“Further, others studies indicated that Tai Chi could improve sleep disorders through increasing functional connections of the sub-regions of DMN, including the medial prefrontal cortex and the medial temporal lobe [38,39]”

**Comment 2:** A few things about this section on Clinical Design and Evaluation are still unclear to me. I read the translated version of the supplement, but the details there are also lacking. One question raised by the supplement was the mention of the McGill Pain Questionnaire and the Hamilton Depression scale as secondary outcome measures. Why were these not mentioned in the main manuscript (unless they were used in a different manuscript)?

Regarding this sentence: “All coaches in the trial were graduates with sports majors and many years of experience with Tai Chi, and they were required to understand the design of the trial and possess basic knowledge of CFS disease.” Were the Tai Chi coaches informed about which group each participant was assigned to (i.e. did they know which participants had CFS and which were healthy volunteers?)? Even if the teaching curriculum was identical for CFS vs HC subjects, coach/trainer awareness of group assignment can bias the teaching and therefore the results. It is also unclear whether CFS and HC participants participated in the same classes together, or whether they were taught separately (i.e. one class contained only CFS and another contained only HC subjects), or whether it was all individual instruction (no groups). Could the authors clarify these questions?

This sentence is still not clear to me: “The decision that the subject who entered the CFS group was made by clinician through consultation’. Is this meant to say something such as “A consulting clinician made each decision about whether each subject would be assigned to the CFS or HC group.”?

Regarding this sentence: “The clinical evaluation both in two groups was conducted using three scale questionnaires: the Fatigue Scale-14 (FS-14) for fatigue symptom assessment(the higher its score, the more serious is the fatigue); Pittsburgh Sleep Quality Index (PSQI)for sleep quality measurement around one month (the higher its score, the lower is the sleep quality); and the MOS 36-item short-form health survey (SF-36) for people’s healthy state evaluation (the higher its score, the healthier is the body)”. Table 1 in the Results shows the baseline values of these scales for each group, but no direct comparison is made. Did the two groups have significant differences in FS-14, PSQI, and SF-36 scores at baseline? What were the cutoffs or thresholds used by the evaluator to determine whether each participant should be assigned to CFS or HC? The table does show p-values for the post-pre differences on each measure within each of the two groups, but did the authors examine whether there was an interaction effect between group and timepoint (i.e. did the CFS group show larger changes on any of these measures than the HC group)?

**Response:** Thanks for your kind reminders.

(1) In the protocol of our research, our team have collected five questionnaires (MPQ, PSQI, HAMD, SF-36, FS-14). However, only three scales were mentioned (PSQI, SF-36, FS-14) in this article. The reasons included:

(a) As a part of our team, we proposed this imaged analysis roadmap (machine learning & fMRI & Tai Chi) to apply for data from the team leader. The other part of data was in another submitted article by my partner.

(b) In this article, we majorly focus on the changes of neuro-image, especially in large-scale brain network. Thus, clinical indicators were only used to express that Tai Chi could improve CFS symptoms rather than to explore all the possible aspects that Tai Chi may affect for CFS. As was descripted in the head of our article, “Chronic fatigue syndrome (CFS)…is characterized by severe fatigue…sleep disturbance…and self-reported impairments in concentration as well as short-term memory [1]”, we think the changes of the three questionnaires we put in our article (including PSQI, SF-36, FS-14) were sufficient to proof the effectiveness of Tai Chi for CFS. For instance, the FS-14 was corresponded to the severe fatigue, the PSQI was corresponded to the sleep disturbance and the SF-36 was corresponded to the self-reported health statement.

(2) Thanks so much, we revised the sentence “All coaches …and they were required to know the design of the trial and the basic knowledge of CFS disease before they took part in this research. On each teaching classes, the subjects in the CFS group or HC group were mixed to be taught but coaches never knew about group information” [Line 137-139].

In our trial, we think that coaches are required to understand our research are their rights to be informed, which cannot be extended to misunderstand that coaches would know much details about processes of our research. Specifically speaking, at the beginning of our trial, we would tell the coach candidates what type of this study and how they would cooperate with us to complete the study. Once they understand what we would do and agree with our study, they would be invited to join us. After that, the only thing that coaches should do is to focus on teaching, and they would never know the group information within their classes.

Besides, HC people and CFS patients were mixed to be taught in each class. In our research, when we recruited sufficient subjects to this trial, we would open a class to teach them for one month. So, HC and CFS were mixed.

(3) Thanks so much. We revised this sentence “A clinician made each decision about whether subject would be assigned to the CFS or HC group” [Line 146-147].

(4) Thanks so much. CFS is a kind of disease that diagnosis majorly depends on patient’s self-report. The including criteria could be found in [Line 115-118] “They had chronic fatigue …and it generally persists or relapses for more than six months…”. Generally, when the person met with this disease description, he would go to our hospital and then be invited to join the trial after clinician evaluation. The clinician evaluation is also majorly relied on asking.

(a) We haven’t made a baseline comparison because we think those two groups were totally different before the intervention. For instance, the CFS group absolutely would have higher scores of FS-14 than HC group, otherwise he would not be recruited into CFS group. Now, for more rigorous in our result, we add the baseline comparison in Table 1 and the analysis description sentence “…and Student’s t-test for independent samples was performed for BMI comparison, questionnaires baseline comparison and questionnaires post-intervention comparison” [Line 224-226].

(b) As we said, CFS is majorly depended on the patients’ self-report, and there still haven’t a clinical indicator to be the diagnostic criteria. Thus, the cutoffs and thresholds would also cannot be captured. The subject would be assigned to CFS or HC majorly depended on his self-report that whether he had a long-tern fatigue syndrome and met with our including criteria.

(c) In this article, detecting the brain networks changes is our main purpose in this article. Thus, we think the t-test’s result would be enough to be the proof of the effectiveness of Tai Chi. Thanks for your kind advise, we now add the repeated measures analysis of variance in clinical measurements in Table 2 and results expression in [Line 248-260]. This helps us a lot, much thanks.

**Comment 3:** Regarding this added sentence: “In the end, all subjects had completed the required exercise time.” If you have data on practice time completed, perhaps you could add that to a table or describe it in the text? If you don’t have specific numbers (minutes, hours, etc) for practice time, which would be preferable but not required, how was it determined that subjects did indeed complete the requirements? Was it verified via the video recordings of all practice sessions or by self-report from participants?

**Response:** Thanks for your kind reminders. We revised this sentence “In the end, all subjects had completed the required exercise times and exercise frequency” to clarify our purpose.

On the process of our trial, each subject was asked to complete the required exercise times (1 hour of teaching classes and 30 minutes of family classes) and exercise frequency (eight times for teaching classes and 20 times for family classes, totally 28 times exercise) [Line 134-135 for teaching classes, 139-140 for family classes]. For teaching classes, we supervised on site and recorded the exercise via live recording. For family classes, we supervised by their video feedback and telephone follow-up [Line 141-142]. Hence, we were confident to say “In the end, all subjects had completed…”. If you asked how we can make sure the truth of each video feedback, we may say that we tried our best to recognize. Anyway, we do have completed the record of each exercise, and we sincerely thought that this sentence desired to be kept, for the sake of our great efforts.

**Comment 4:** Thank you for adding the details, very helpful!

**Response:** Thank you.

**Comment 5:** Great, thanks for the clarification about your smoothing process.

**Response:** Thank you.

**Comment 6:** The changes look good.

**Response:** Thank you.

**Comment 8:** The figure is a bit clearer now. However, one question remains about the significance of the results. You have mentioned in other parts of the paper that strict Bonferroni correction was applied. In the case of the partial correlation presented in Figure6a, the p-value is 0.28. Even if that correlation and the one shown in 6b were the only two partial correlation tests you ran (were there others?), the corrected Bonferroni p-value for two tests would be 0.025, making the first one non-significant. Unless I am missing something, what is making you confident that this is a truly significant result?

**Response:** Thanks for your kind reminders.

Well, actually, these two (Figure 6A and Figure 6B) are not under the same hypothesis. We put these two together just for convenience to display. However, we now realize that this action could confuse the readers. Sorry for that.

Partial covariance analysis was the further exploration for our main results. We may first wonder whether there was correlation between DMN and LFPN, and we got the answer (Figure 6B). Then, we detected the correlations between networks (DMN / LPFN) and clinical measurements (FS-14 / PSQI / SF-36), and we got the answer (Figure 6A). We thought these two results were under the different hypotheses, so we did not perform the Bonferroni correction.

However, not only did you propose the potential false-positive in Figure 6A, but we also found the inexplicable part between Figure 6A and our others results. To consider the data’s integrity and result’s consistency, we haven’t deleted Figure 6A in the previous manuscript. Thanks for your carefulness, we choose to delete the Figure 6A and all the explanation sentences about Figure 6A in Discussion.

**Additional comments:**

In some newly added lines in the Discussion, it says “we found that the increases of functional connections between the DMN and other networks may also correlate to the improvements of fatigue and sleep disorder (Table 1).” Table 1 does not show associations between DMN functional connectivity and fatigue/sleep disorder symptoms, so this reference might need to be updated?

**Response:** Thanks for your kind reminders. We revised this sentence “Differently, we found that the increases of functional connections between the DMN and other networks could be the reasons of improvements of fatigue and sleep disorder (Tables 1-4)” [Line 365-367].

In our trial, the FCs of DMN and other networks increased and clinical measurements improved, and we thought these could be the mechanism of Tai Chi treating CFS. However, previous literature only focused on the sub-regions within DMN and the improvements of clinical symptoms. That was why we used “Differently” in here. Thanks for your reminders, we have realized that it was better to use the “reason” rather than the “correlate” to express our meaning accurately. Hence, we revised our description. Thanks again, your kind advise helped me a lot.

On Line 392, you say that your results “proves the effectiveness of Tai Chi exercise for CFS alleviation.” “Proves” is a very strong word and I think much more work is needed before we have “proof” that Tai Chi is effective for CFS! I would use more cautiously optimistic language here. Even from a non-fMRI data perspective, you have not shown an interaction effect demonstrating that the symptom improvements after Tai Chi for the CFS group were significantly greater than those seen in the HC group. Unless you can show that, I would not use this kind of language in the paper, and even then I would be more cautious.

**Response:** Thanks for your kind reminders. We changed this word with “illustrates” [Line 407]. Thanks again, your carefulness helped me a lot.
